# Asia at the Epicenter of the Global Cardiometabolic Shift

**DOI:** 10.1016/j.jacasi.2026.01.001

**Published:** 2026-03-03

**Authors:** Shaun Khanna, Gary C.H. Gan, Andrew P. Sindone, Jasper Tromp, Javed Butler, Roger Foo, Nitesh Nerlekar, Aditya Bhat

**Affiliations:** a The George Institute for Global Health, UNSW Sydney, NSW, Australia; b Baker Heart and Diabetes Institute, NSW, Australia; c Department of Cardiology, Blacktown Hospital, NSW, Australia; d Department of Cardiology, Westmead Hospital, NSW, Australia; e Department of Cardiology, Concord Repatriation Hospital, University of Sydney, NSW, Australia; f Saw Swee Hock School of Public Health and National University of Singapore and National University Health System, Singapore; g University Medical Centre Groningen, Department of Cardiology, University of Groningen, Groningen, the Netherlands; h Baylor Scott and White Research Institute, Dallas, Texas, USA; i University of Mississippi, Jackson, Mississippi, USA; j Cardiovascular Research Institute, National University Heart Centre, Singapore, Cardiovascular Metabolic Disease Translational Research Programme, Yong Loo Lin School of Medicine, National University of Singapore, Singapore; k Victorian Heart Institute, Victorian Heart Hospital, Clayton, VIC, Australia

**Keywords:** cardiometabolic disease, diabetes, hypertension, obesity

## Abstract

Cardiometabolic disease has become the dominant noncommunicable health challenge of the 21st century, with its burden increasingly centered in Asia. Rising obesity, type 2 diabetes mellitus, hypertension, and dyslipidemia now account for more than one-third of cardiovascular mortality. This escalation reflects the interaction of biological susceptibility with rapid urbanization, digitalized food environments, physical inactivity, and persistent tobacco exposure. Health-system limitations, including low diagnosis rates, poor risk-factor control, and uneven access to essential therapies, further amplify vulnerability across South, East, and Southeast Asia. Environmental pressures such as air pollution and extreme heat compound these risks, while migrant data illustrate how biological predisposition is magnified in obesogenic settings. This review synthesizes evolving epidemiology, biological diversity, behavioral and environmental drivers, and health-system gaps shaping cardiometabolic risk across Asia. It also outlines policy and therapeutic strategies, including strengthened primary care, prevention-focused interventions, and emerging therapeutics needed to reduce cardiometabolic disease in the region.

## Highlights

• Cardiometabolic disease is increasing fastest in Asia due to biological vulnerability and health-system constraints.
• Metabolic risk develops at lower BMI in Asian populations, limiting the use of Western risk thresholds.
• Life-course prevention and stronger primary care are central to managing cardiometabolic multimorbidity.
• Progress will depend on fair access to effective therapies and risk tools tailored to Asian populations.

Cardiometabolic disease and obesity have become the defining health crisis of the 21st century. Aligned with the American Heart Association cardiovascular–kidney–metabolic framework, cardiometabolic disease is defined as the interconnected continuum of excess or dysfunctional adiposity, dysglycemia and type 2 diabetes, hypertension, and atherogenic dyslipidemia.^1^ This continuum gives rise to major downstream cardiovascular sequelae, including atherosclerotic cardiovascular disease, stroke, and heart failure, and is closely linked to the burden of chronic kidney disease.

Across the Asia–Pacific region, cardiometabolic disease now accounts for more than a third of deaths and the majority of premature mortality in adults >40 years of age.^2^ In contrast to many Western regions, Asia is experiencing accelerating metabolic deterioration, despite current guideline and therapeutic advances in prevention and treatment.^3^ In 2024, the Asia–Pacific accounted for 4.8 billion people, a number expected to exceed 5.2 billion by 2050, with the most dramatic expansion occurring in South and South-West Asia.^4^ These demographic forces amplify the region’s vulnerability, placing Asia at the epicenter of the global cardiometabolic transition.^5^

There is growing recognition that Asia’s cardiometabolic burden is shaped by the presence of biological susceptibility, rapid urbanization, accelerating digitalization, and uneven health-system capacity. Conventional cardiovascular risk–prediction tools, including the American College of Cardiology/American Heart Association Pooled Cohort Equations, were largely derived and calibrated in Western populations and do not adequately capture the heterogeneity of cardiometabolic risk across Asia.^6^ Miscalibration has been demonstrated not only in Korean cohorts,^7^ but across multiple East, South, and Southeast Asian populations,^8^ where risk is frequently underestimated or overestimated depending on ethnicity, age, and baseline metabolic profiles.

For example, the SCORE2 (Systematic Coronary Risk Evaluation 2) miscalibrates risk in several Asia–Pacific settings, prompting development of region-specific approaches.^8^ The American Heart Association’s PREVENT^9^ Equations represent an important advance in cardiovascular risk prediction; however, current validation is largely based on Asian individuals living in Western settings. Consequently, PREVENT may not fully capture the distinct cardiometabolic exposures of populations residing in Asia, underscoring the importance of nuanced risk characterization across heterogeneous Asian populations. This review synthesizes the evolving epidemiology, biological diversity, behavioral and structural drivers, and health-system challenges shaping cardiometabolic risk across Asia (Central Illustration). We aim to provide a contemporary framework to guide prevention, risk assessment, and policy across the world’s most populous and increasingly most vulnerable region.

**Central Illustration:**
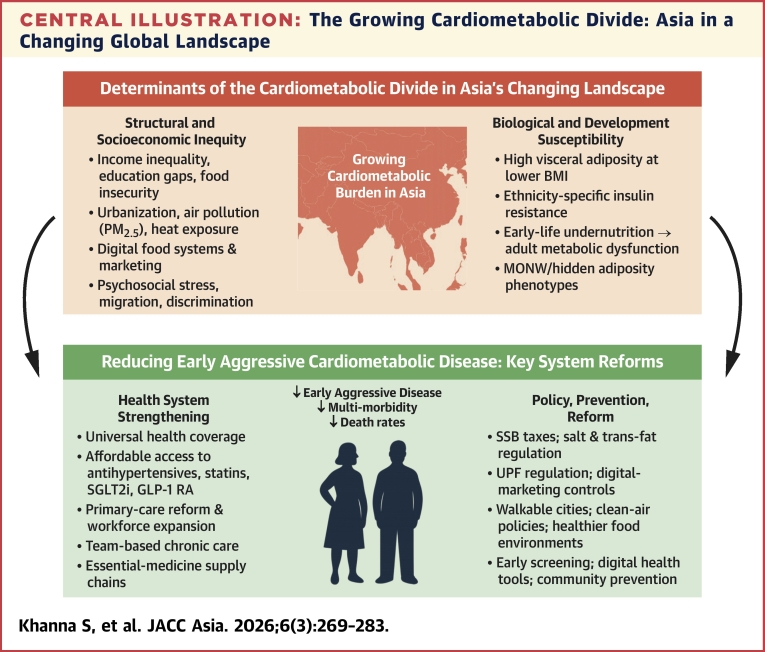
The Growing Cardiometabolic Divide: Asia in a Changing Global Landscape This illustration summaries how structural inequities and obesogenic environments interact with biological and developmental susceptibility to drive early, aggressive cardiometabolic disease across Asia. Health-system strengthening and population-level prevention are essential to reduce multimorbidity and premature mortality. BMI = body mass index; GLP-1 RA = glucagon-like peptide-1 receptor agonist; MONW = metabolically obese, normal-weight; PM_2.5_ = particulate matter ≤2.5; SGLT2 = sodium–glucose cotransporter-2; SSB = sugar-sweetened beverages; UPF = ultra processed foods.

## Global Patterns and Shifting Asian Epidemiology

Global cardiovascular mortality linked to metabolic risk factors has undergone significant shifts over the past three decades. Analyses from the Global Burden of Disease study show that between 1990 and 2021, the global age-standardized mortality rate attributable to metabolic risks decreased by approximately 1.3% per year.^10^ High–sociodemographic index countries achieved even steeper annual reductions approaching 3%, largely through widespread antihypertensive therapy, lipid management, and improved preventative care. By contrast, low–sociodemographic index nations, particularly in South Asia, experienced stagnation or marginal increases in metabolic mortality despite comparable risk exposures.^10^, ^11^, ^12^

The absolute number of deaths attributable to metabolic factors have increased by more than 60% over the same period.^10^ This reflects population growth, ageing, and persistent inequities in health access and risk-factor control. Asia sits squarely at the epicenter of this transition. Whereas in 1990 nearly 60% of cardiometabolic deaths occurred in high-income regions, by 2021 this proportion had decreased to <30%.^13^ Today, >80% of diabetes-related mortality and nearly three-quarters of hypertension-related cardiovascular deaths occur in low- and middle-income countries (LMICs), many of which are in Asia.^14^

Within these settings, rapid dietary and lifestyle shifts compound the epidemiologic shift. Increasing consumption of processed and ultraprocessed foods, refined carbohydrates, and energy-dense diets, combined with increasingly sedentary behavior, has accelerated the rise of metabolic syndrome across the region.^15^ The risk-factor landscape continues to evolve rapidly: projections indicate that type 2 diabetes prevalence in Asian countries will more than double between 2000 and 2035.^16^ Hypertension remains the dominant driver of cardiovascular mortality, yet control rates remain alarmingly low. Data from the HOPE Asia Network show that across 11 Asian countries, <20% of individuals with hypertension achieve adequate blood-pressure control, contributing substantially to the regional cardiovascular burden.^17^ As shown in Table 1, the Asia–Pacific region exhibits substantial heterogeneity in behavioral and metabolic risk factors.

**Table 1:** Cardiometabolic Risk Factors Across Asia–Pacific Countries

Country	CVD Mortality (% of Total Deaths)	Physical Inactivity (%)	Tobacco Use (%)	Salt Intake (g/d)	Raised BP (%)	Diabetes (%)	Obesity (%)	Dyslipidemia (%)
Australia	28	32	13	8	19	7	30	33
Bangladesh	30	26	23	8	22	8	3	—
Cambodia	24	10	17	11	21	6	4	—
China	43	15	27	12	21	9	30	12
Fiji	33	33	22	9	20	18	7	—
India	27	22	11	9	26	8	6	30
Indonesia	27	38	38	12	23	7	7	—
Japan	34	20	20	10	23	6	4	17
Laos	27	14	22	12	19	6	5	—
Malaysia	35	43	20	12	23	10	15	45
Myanmar	31	37	24	11	21	7	5	—
New Zealand	28	10	14	9	17	5	32	—
Philippines	23	38	22	15	16	9	7	—
Singapore	34	38	15	10	24	8	9	17
South Korea	29	37	22	11	26	10	10	14
Sri Lanka	31	29	14	10	24	9	9	—
Thailand	22	24	20	14	22	11	11	—
Tonga	33	20	27	7	25	22	42	—
Vietnam	31	24	24	12	23	5	3	—

Values are n (%).

Country-level estimates of key behavioral and metabolic risk factors, including proportion of cardiovascular mortality, physical inactivity, tobacco use, daily salt intake, raised BP, diabetes prevalence, obesity prevalence, and dyslipidemia. Approximates extracted from *Tackling cardiometabolic risk in the Asia Pacific region* (Li JJ, Yeo KK, Tan K, et al. *Am J Prev Cardiol*. 2020;4:100096). These data demonstrate striking heterogeneity across the Asia–Pacific region and underscore the diverse cardiometabolic profiles shaping population health.

BP = blood pressure; CVD = cardiovascular disease.

## Determinants of Cardiometabolic Disease

### Biological heterogeneity

Biological and ethnic differences substantially influence cardiometabolic risk across Asia. Variations in adipose distribution, insulin sensitivity,^18^ and lipid metabolism mean that Asian populations develop metabolic disease at lower body mass index (BMI) cutoffs than Europeans.^19^ South Asians exhibit insulin resistance and type 2 diabetes mellitus at BMIs 3-5 kg/m^2^ lower than Europeans, often at thresholds as low as 21-23 kg/m^2^.^20^ East Asians, in contrast, accumulate disproportionately more visceral and hepatic fat despite relatively low BMI or waist circumference.^21^ Across the region, lower lean mass coupled with higher visceral and intra-abdominal fat for any given BMI contributes to earlier and long-lasting metabolic vulnerability,^22^ especially in Asian populations.^23^ Intrinsic differences in insulin sensitivity and energy metabolism,^24^ including greater insulin resistance at lower adiposity and reduced β-cell reserve,^25^ predispose Asian populations to earlier cardiometabolic disease despite relatively low BMI. Notably, insulin resistance in nonobese Asian individuals has been linked to altered amino-acid metabolism rather than circulating lipids or inflammatory markers,^26^ underscoring qualitative metabolic vulnerability.

Genetic susceptibility contributes to these phenotype differences and helps explain why elevated cardiometabolic risk persists even after migration to Western environments.^27^ South Asian–specific genomic studies suggest limited transferability of European-derived central obesity loci, indicating that inherited determinants of waist-hip ratio and visceral adiposity differ by ancestry.^28^ Variants near genes involved in neuroendocrine energy regulation (ie, MC4R) and adipose tissue biology have been associated with central adiposity in Indian and East Asian populations,^29^ whereas candidate loci such as NID2 and HECTD4 have been linked to adipogenesis, visceral fat expression, and diabetes risk.^30^ Although individual loci explain only a small proportion of phenotypic variance, these findings support a polygenic predisposition to metabolically adverse fat distribution that interacts with environment and persists across life-course and migration.

Population-based data reinforce these compositional differences. In the Chennai Urban Rural Epidemiology Study, optimal thresholds for detecting ≥2 cardiometabolic risk factors were approximately 23 kg/m^2^ for BMI and 87/82 cm for waist circumference in men and women, significantly lower than conventional “obesity” cut-offs.^31^ These findings underscore the limitations of BMI-centric definitions of obesity and highlight the need for recalibrated, region-specific anthropometric thresholds.^32^ They also emphasize that cardiometabolic risk is driven not only by adipose tissue quantity, but also by its quality, including distribution, inflammatory profile, and endocrine activity.

The clinical expression of cardiometabolic disease also differs by socioeconomic context. In high-income settings, disease presentation tends to reflect chronic atherosclerosis and heart failure with preserved ejection fraction,^33^ whereas in low-income regions, cardiomyopathy, stroke, renal disease, and earlier multisystem involvement predominate.^33^ Emerging data further show that vulnerability begins early. A study of adolescents living in Karachi slums reported a metabolic syndrome prevalence of 16.7%, driven by low high-density lipoprotein, high triglycerides, and elevated fasting glucose, underscoring the early-life emergence of metabolic risk in marginalized South Asian youth.^34^ Similarly, overweight and obese Korean adolescents showed markedly higher clustering of cardiometabolic risk factors, with obesity increasing the odds of having ≥1, ≥2, and ≥3 abnormalities by 2.8-, 3.8-, and 4.8-fold, respectively, compared with normal-weight peers.^35^

**Figure 1:**
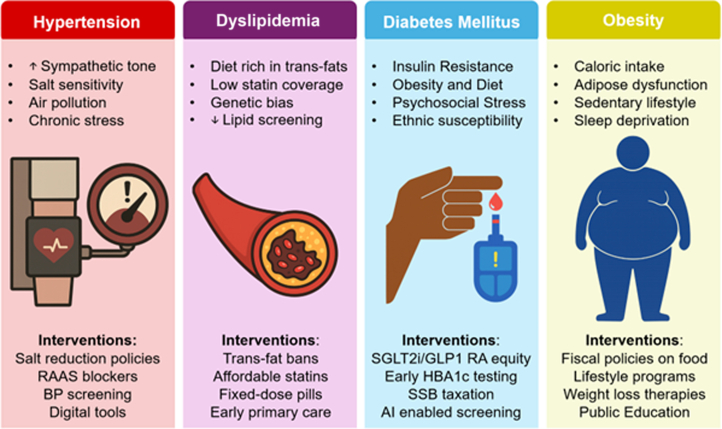
Determinants and Targeted Interventions Across the Cardiometabolic Spectrum in Asia This figure summarizes key determinants and policy interventions for 4 major cardiometabolic risk factors: hypertension, dyslipidemia, diabetes mellitus, and obesity. Corresponding interventions span population-level and clinical strategies: salt reduction and BP screening for hypertension; trans-fat bans and affordable statin access for dyslipidemia; equitable access to SGLT2 inhibitor and GLP-1 receptor agonist therapies, early diabetes screening, and taxation of SSB for diabetes; and fiscal food policies, structured lifestyle programs, and weight-loss therapies for obesity. Together, these measures highlight integrated pathways to reduce global cardiometabolic disease burden. BP = blood pressure; GLP-1 = glucagon-like peptide-1; HbA1c = glycated hemoglobin; RAAS = renin-angiotensin-aldosterone system; SGLT2 = sodium–glucose cotransporter-2; SSB = sugar-sweetened beverages.

Epigenetic and developmental factors add an additional layer of complexity. Low birth weight and early-life undernutrition, still highly prevalent in many low- and middle-income Asian countries, predispose individuals to central adiposity and metabolic dysregulation when exposed to caloric abundance later in life.^36^ This “mismatch” produces metabolically obese but normal-weight phenotypes,^37^ a pattern increasingly evident across South and East Asia (Figure 1).^38^

### Behavioral and lifestyle factors

Industrialization has reshaped food environments across Asia more rapidly than in any other region. Between 2000 and 2020, per-capita consumption of sugar-sweetened beverages increased by more than 50%, while intake in many Western countries plateaued or decreased.^39^ Modelling estimates attribute more than 1.1 million cardiovascular events each year to sweetened-beverage intake alone.^40^ Salt consumption remains nearly double World Health Organization (WHO) recommendations worldwide and contributes an estimated 1.8 million deaths annually.^41^ These dietary patterns initiate a cascade of metabolic injury that accelerates atherosclerotic progression,^42^ and underlies the cardio–renal–metabolic disease spectrum now dominating clinical practice across Asia.^43^

**Figure 2:**
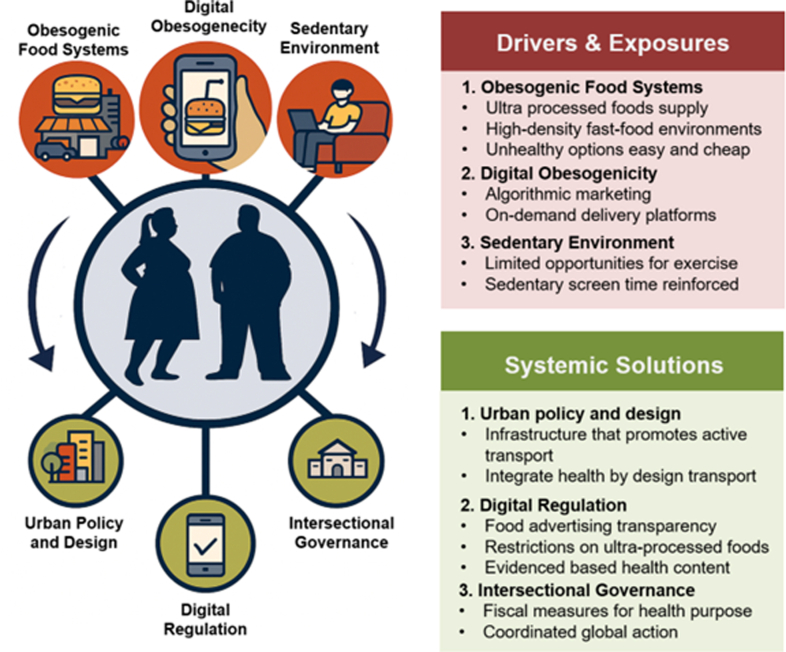
A Syndemic Framework for Obesogenic Environments Obesogenic food systems, digital marketing environments, and sedentary lifestyles interact to reinforce weight gain and metabolic risk. These upstream drivers operate through ultraprocessed food availability, algorithmic advertising, and limited opportunities for physical activity. System-level solutions—urban policy and design, digital regulation, and intersectional governance—offer coordinated strategies to reshape environments, improve health behaviors, and reduce population-level obesity risk.

Asian populations are particularly vulnerable to these dietary shifts. Obesity in the region reflects a systems-level biological vulnerability compounded by modern food systems, digital marketing, sedentary lifestyles, and entrenched social inequities.^44^ These reinforcing pressures accelerate the onset and severity of cardiometabolic disease, often at younger ages than observed elsewhere. Cohort studies from China, Singapore, and India consistently show that diabetes mellitus develops 5-10 years earlier and at lower BMI thresholds than in Western populations, driven by greater visceral adiposity, reduced beta-cell reserve, and rising exposure to ultraprocessed foods (Figure 2).^45^

Consistent with these early and sustained behavioral exposures, longitudinal data now demonstrate that excess adiposity in early life and early adulthood confers durable cardiovascular risk that is not fully reversible by later lifestyle modification.^46^ In the China Kadoorie Biobank, higher BMI during early adulthood was associated with a monotonic increase in incident cardiovascular disease^47^ over more than a decade of follow-up. Notably, excess risk was evident from BMI as low as 23 kg/m^2^, well below conventional Western obesity thresholds, and favorable midlife lifestyle behaviors did not materially attenuate these associations.^47^ Alarmingly, deficits in cardiovascular health are already established in childhood. In a national study of more than 15,000 Chinese children and adolescents, fewer than 2% met criteria for ideal cardiovascular health,^48^ driven primarily by poor physical activity and diet. Collectively, these data establish childhood and early adulthood as a critical, nonrecoverable window for obesity prevention in Asian populations, highlighting the urgency of life-course interventions implemented before metabolic disease becomes established.

Physical inactivity is increasing fastest in low-income regions, including South-East Asia, driven by urbanization, outdoor environments, and widespread motorization.^49^ Sedentary time ≥9 h/d is associated with 25%-40% higher odds of multiple metabolic comorbidities.^50^ Across Asia, rapid decreases in walking and cycling have markedly reduced daily energy expenditure.^51^

Tobacco use further compounds these risks. Although global smoking rates are decreasing, tobacco use remains heavily concentrated among men in lower-income Asian countries.^52^ In Indonesia, Bangladesh, and China, male smoking prevalence consistently exceeds 40%-50%, contributing to some of the world’s highest rates of tobacco-attributable cardiovascular mortality.^53^^,^^54^

These behaviors rarely occur in isolation. A pooled analysis of WHO STEPS surveys from 10 South and Southeast Asian countries shows substantial clustering: more than one-third of adults have multiple metabolic risk factors, and >70% have 2 or more behavioral risks, including poor diet, inactivity, and smoking.^55^ This clustering of risks sharpens disparities between those with supportive environments and those without.

### Social and economic factors

Economic development alone does not guarantee improved cardiometabolic health. Countries with similar gross domestic product often diverge markedly in cardiovascular and metabolic mortality because of differences in education, inequality, and investment in primary health care (PHC) systems.^56^ Cross-national analyses show that the most efficient countries, those achieving the lowest metabolic mortality for their level of development, outperform less efficient peers by up to 40%.^10^ This divergence is widening across Asia as urbanization reshapes both risk and access. Urban residents often benefit from greater access to healthcare but face higher exposure to energy-dense diets, air pollution, and psychosocial stress. In contrast, rural populations experience underdiagnosis, limited chronic disease services, and fewer opportunities for follow-up care.^57^

At the same time, targeted population-level programs show that structural interventions can meaningfully shift metabolic risk. China’s national public-health initiative, for example, demonstrated that community-based hypertension screening in low-income rural regions produced an additional 6.3 mm Hg reduction in systolic blood pressure (BP) over 2 years compared with unscreened communities.^58^ In Nepal, a community-health-worker–led home-visit program modestly improved BP, physical activity, salt intake, and hypertension knowledge, underscoring the feasibility of decentralized preventive care in low-resource settings.^59^

Psychosocial and social-structural forces further shape metabolic vulnerability. Studies consistently link perceived discrimination and chronic social exclusion to higher rates of metabolic syndrome,^60^ mediated by neuro-endocrine^61^ and inflammatory activation that increases cortisol, catecholamine exposure, ectopic fat deposition, and glucose dysregulation.^62^ Importantly, this pathway is modifiable: a participatory, community-mobilization program in rural Bangladesh improved diabetes knowledge and produced modest reductions in hypertension and abdominal obesity, demonstrating that educational and psychosocial interventions can attenuate metabolic risk.^63^

Finally, acute societal shocks expose the fragility of chronic-disease care across Asia. During the COVID-19 pandemic, widespread interruptions in medication supply and clinic attendance resulted in significant treatment lapses.^64^ The COVID-19 pandemic was associated with worsening metabolic parameters, including higher BMI, BP, lipids, and metabolic syndrome prevalence, in individuals with cardiometabolic disorders (Table 2).^65^

**Table 2:** Structural Determinants of Cardiometabolic Inequity in Asia

Determinant	Key Drivers in Asia	Illustrative Data/Mechanisms	Contribution to Inequity	Example Asian Region(s)	Potential Interventions
Socioeconomic disadvantage	Income inequality, education gaps, food insecurity	Adults with low education have 1.5-2× higher cardiometabolic mortality; urban poor have highest exposure to ultraprocessed foods	Limits access to healthy diets, preventive care, and health literacy	India, Indonesia, Philippines	Conditional cash transfers; healthier food subsidies; expansion of secondary education
Urbanization & built environment	Dense food swamps, motorization, limited green space	SSB intake in Asia ↑ 50% (2000-2020); urban PM_2_._5_ among world’s highest	Drives obesity, diabetes, and hypertension in rapidly urbanizing areas	China, Vietnam, Bangladesh	SSB taxes; walkable-city design; clean-air standards; zoning to reduce unhealthy food density
Healthcare access & workforce capacity	Out-of-pocket costs, fragmented care, shortages of clinicians	Hypertension control <20% in several South/Southeast Asian countries; physician density 0.6-0.9 per 1,000 in South Asia	Preventable mortality due to late diagnosis and undertreatment	South Asia, Southeast Asia	Universal health coverage; essential-medicine supply chain reform; task-sharing with nurses/CHWs; digital decision-support tools
Psychosocial & structural stressors	Economic insecurity, migration, discrimination, conflict zones	Chronic cortisol elevation → ↑ visceral fat, ↑ BP, ↑ glucose	Sustains biological risk in vulnerable groups	Sri Lanka, Myanmar, migrant workers across Gulf & Asia	Social protection programs; workplace protections; community mental-health services
Biological susceptibility	Ethnic variation in adiposity and insulin sensitivity	South Asians develop T2D at BMI 21-23 kg/m^2^; East Asians have higher visceral fat for same BMI	Standard BMI/WC thresholds underidentify high-risk Asian individuals	South Asia, East Asia	Ethnic-specific cut-points; regional risk scores; inclusion of Asia in genomic/imaging studies
Early-life programming	Maternal under-nutrition, low birth weight, rapid early weight gain	Low birth weight → ↓ β-cell reserve + ↑ visceral adiposity in adulthood	Intergenerational transmission of metabolic risk	India, Nepal, Pakistan	Maternal nutrition; antenatal screening; early-childhood growth monitoring
Policy & governance gaps	Underinvestment in NCD prevention, weak regulation of industry	<2% of global health aid allocated to NCDs; weak regulation of UPFs and digital marketing	Structural neglect perpetuates disparities in metabolic risk	Many LMICs in Asia	Fiscal measures (UPF taxes), regulation of digital food marketing, stronger NCD financing and accountability frameworks

This table summarizes the key socioeconomic, environmental, biological, and health-system factors that drive heterogeneity in cardiometabolic risk across Asian populations. It highlights how these structural conditions interact to amplify vulnerability and outlines regionally relevant policy and clinical interventions to reduce inequity.

BMI = body mass index; CHW = community health worker; NCD = non-communicable disease; PM_2.5_ = particulate matter ≤2.5; SSB = sugar-sweetened beverage; T2D = type 2 diabetes; UPF = ultra processed foods; WC = waist circumference; other abbreviations as in Table 1.

### Environmental and systemic exposures

Environmental change is increasingly interwoven with cardiometabolic health, and this relationship is especially visible across Asia. Air pollution contributes to an estimated 6.7 million deaths each year, nearly one-third of which are from cardiovascular causes, with Asian countries experiencing the highest global concentrations of particulate matter ≤2.5 μm.^66^ Fine particulates trigger systemic inflammation, endothelial dysfunction, and insulin resistance, creating a direct pathway between environmental exposure and cardiometabolic injury.^67^ Accordingly, long-term exposure to particulate matter ≤2.5 μm, particulate matter ≤10 μm, and nitrogen dioxide have been shown to be associated with a higher prevalence of metabolic syndrome among Chinese children and adolescents.^68^

Extreme heat adds another emerging threat, particularly in regions closer to the equator, where baseline temperatures are higher, and heat exposure is more prolonged. In 2021, high temperatures were responsible for more than 88,000 ischemic heart disease deaths and more than 2 million disability-adjusted life-years across Asia,^69^ with heat-attributable cardiovascular mortality increasing at approximately 1.6% per year.^70^ These climatic stresses disproportionately affect older adults, laborers, and urban populations with limited access to cooling and green space.

Policies that prioritize industrial growth and mobility often accelerate dietary shifts toward processed foods and reduce opportunities for daily physical activity.^71^ The expansion of motorized transport reduces active commuting and contributes to rising sedentary behavior, amplifying cardiometabolic risk at a population level.^72^ Conversely, cities that invest in walkable neighborhoods, clean air, and accessible fresh foods consistently show lower rates of obesity and hypertension, even after adjusting for socioeconomic status.^73^

### Clinical complexity

As cardiometabolic risk factors accumulate, they increasingly cluster rather than occur in isolation, a pattern especially prominent across Asia. Population-level screening shows that up to 10% of older adults now present with 3 or more metabolic conditions.^74^ Large-scale data involving more than 4 million adults demonstrate that 2.7% have concurrent diabetes mellitus, hypertension, and obesity, with prevalence increasing to nearly 10% among individuals older than 60 years.^75^ In hospital cohorts, more than half of patients admitted with coronary artery disease carry at least 2 additional metabolic diagnoses, and each added condition roughly doubles the hazard of major adverse cardiovascular events.^76^

Clinically, this multimorbidity presents as earlier-onset heart failure, a greater stroke burden, and rapid progression of vascular and renal disease across Asian populations.^77^ Yet most health systems in the region remain oriented toward single-disease management, resulting in fragmented care and missed opportunities for coordinated risk reduction. Factors such as polypharmacy, suboptimal follow-up, and financial barriers further undermine control of BP, glucose, and lipids.^78^ In LMICs, where out-of-pocket expenditure is common, patients with multiple chronic conditions frequently ration medications or forgo treatment altogether, compounding this vulnerability.^79^

## Migration as a Natural Experiment

The growing Asian migration provides a natural experiment illustrating how biological susceptibility interacts with environmental and social context. Asian migrants now represent the fastest-growing immigrant group in the United States^80^ and a major minority population across Europe,^81^ yet they consistently exhibit higher rates of metabolic disease than non-Hispanic White adults. In the National Health and Nutrition Examination Survey, diabetes mellitus prevalence reaches 19.1% in Asian Americans compared with 12.1% in White adults, with South Asian, Filipino, and Vietnamese groups carrying the highest burden.^82^ UK Biobank data similarly show that South Asians living in the United Kingdom have 2- to 4-fold higher risks of type 2 diabetes mellitus, coronary artery disease, and chronic kidney disease relative to Europeans.^83^

Acculturation strongly modifies this risk profile. Foreign-born Asian adults and recent immigrants (<5 years in the host country) have significantly higher diabetes risk than those born locally, suggesting rapid metabolic consequences of adopting Western dietary patterns and lifestyles.^84^ Structural barriers, including language challenges, limited preventive care access, and experiences of discrimination, further compound vulnerability. Together, these migrant patterns underscore a critical insight: inherent biological predisposition becomes magnified when combined with obesogenic environments and social disadvantage.

## Health System Capacity and Access Gaps

The capacity to detect, treat, and manage cardiometabolic disease varies profoundly across countries, with Asia illustrating some of the widest global disparities. High-income regions have achieved near-universal access to essential therapies and have integrated guideline-based cardiovascular prevention into routine PHC.^85^ In contrast, in many low-income Asian countries, fewer than half of adults with hypertension are even aware of their diagnosis, and fewer than 1 in 5 achieve adequate blood-pressure control.^85^

Additionally, across Asia, PHC systems are frequently structured for acute, episodic illness rather than for the sustained management of chronic cardiometabolic conditions.^86^ PHC financing in many low- and middle-income Asian countries remains largely out-of-pocket, with weaker integration for noncommunicable care, resulting in delayed diagnosis and continuity gaps in management.^87^ Similarly, analyses of primary care across Asia point to fragmented purchasing, limited investment in preventive services, and uneven provider networks.^88^

Access to core medications remains uneven. As of 2023, only 59% of LMICs ensured continuous public-sector availability of all 3 foundational drug classes, blood-pressure–lowering agents, statins, and metformin.^89^ Where universal health coverage is incomplete, chronic disease management frequently imposes catastrophic expenditure, forcing patients to ration medications or discontinue treatment altogether, a recurrent challenge in South and South-East Asia.^90^

Basic strengthening of population-wide hypertension control alone could avert an estimated 4.5 million premature deaths per decade in LMICs.^91^ Yet implementation is constrained by severe workforce shortages: physician densities range from only 0.6-0.9 per 1,000 in Bangladesh, Bhutan, India, Indonesia, and Nepal, and 1.2 per 1,000 in Sri Lanka, among the lowest globally.^92^ Task-shifting to community health workers and the use of digital decision-support tools have demonstrated measurable improvements in metabolic risk-factor control, but current coverage remains insufficient to meet population needs.^93^

Fragmentation of care further limits effectiveness. Diabetes mellitus, hypertension, and dyslipidemia are commonly managed in isolated clinical pathways, leading to missed opportunities for integrated cardiometabolic risk reduction.^94^ Team-based chronic-disease models that incorporate nurses, pharmacists, and community health navigators have consistently demonstrated superior control of BP, glycated hemoglobin, and lipids compared with physician-only care.^95^ Such multidisciplinary approaches are increasingly essential as multimorbidity becomes the dominant clinical reality across Asia.

Beyond population-level prevention and strengthened primary care, referral-based cardiometabolic specialty clinics can serve as escalation hubs within shared-care models.^96^ Appropriate referrals include early-onset/severe disease, multimorbidity (≥2 of diabetes mellitus, hypertension, obesity, chronic kidney disease, atherosclerotic cardiovascular disease), treatment-resistant risk factors (for example BP uncontrolled on ≥3 agents, refractory hyperglycemia, persistently elevated low-density lipoprotein cholesterol despite therapy), or progressive end-organ injury/high-risk phenotypes (recurrent events, rapidly progressive chronic kidney disease, suspected familial hypercholesterolemia, severe obesity requiring advanced pharmacotherapy, complex polypharmacy).^97^^,^^98^ Delivery in constrained systems can rely on task-sharing; for example, nurse practitioners can run protocolized titration and monitoring (BP/lipids/glycemia, renal function/electrolytes) with escalation triggers,^99^ while pharmacists lead medication reconciliation, adherence, and adverse-effect surveillance.^100^ These pathways are all supported by algorithms and physician oversight.

## Therapeutics and Policy Innovation

### Policy and prevention strategies

Closing the global divide will depend on coordinated action across 4 fronts: prevention, healthcare delivery, social reform, and global leadership.^101^ Global frameworks provide practical scaffolding for these reforms. The WHO Global Strategy on Diet, Physical Activity and Health^102^ emphasizes population-level policies to improve diet quality and physical activity and to reshape obesogenic food environments. In parallel, the WHO HEARTS package operationalizes protocol-based, primary care delivery for cardiovascular risk-factor control through standardized treatment pathways, reliable access to essential medicines, and monitoring systems.^103^ Similarly, the WHO ROOTS approach further complements these strategies by prioritizing upstream, community-level and life-course prevention of obesity and related cardiometabolic risk,^104^ which is particularly relevant for LMIC settings across Asia.

Health-economic policies such as sugar-sweetened beverage taxes have proven effective, with a 10% price increase typically reducing consumption by 8%-10%.^105^ Trans-fat bans in more than 60 countries now prevent an estimated 300,000 deaths each year.^106^ In the United Kingdom, national salt-reduction policies lowered mean BP by 3 mm Hg and reduced cardiovascular deaths by nearly 40% within a decade.^107^ These examples show how legislation, when adapted to Asian contexts, can shift population health in measurable ways.

Cardiometabolic prevention must also strengthen primary care. In many low-income Asian countries, primary care capacity is further constrained by how it is financed; in settings like Lao PDR^108^ and Cambodia^109^ where primary care is largely paid for out-of-pocket and provided by variably accredited clinics, quality control and continuity remain major obstacles to effective cardiometabolic management.

Early metabolic screening, digital health tools, and community-based interventions, such as BP kiosks, mobile glucose testing, and remote medication reminders, have shown measurable gains in detection and adherence.^110^ The WHO HEARTS program provides a scalable model of protocol-based hypertension and diabetes management, supported by reliable drug supply and community follow-up.^103^ Countries such as Thailand have achieved control rates >50%, demonstrating that success is possible even in middle-income settings.^111^ Complementary digital strategies also show promise: a systematic review of 8 Indian studies found that mobile-health interventions (SMS education, digital platforms) improved BP, glycated hemoglobin, fasting glucose, treatment adherence, and patient satisfaction.^110^

Regional guidance efforts also exist for strategic prevention. The Asia Pacific Cardiometabolic Consortium has developed consensus recommendations to unify secondary prevention strategies after myocardial infarction across the Asia–Pacific region.^112^ However, these recommendations focus on postevent management rather than population-level screening or primary prevention, and many Asian countries continue to rely on Western-derived guidelines for cardiovascular risk assessment and prevention.

Diet-related policies remain central. Sodium intake routinely exceeds 10 g/d in China, Japan, and several South and Southeast Asian countries. Modelling suggests that a 1 g/d reduction in salt intake in China alone could prevent up to 9 million cardiovascular events by 2030.^113^ Evidence from a 20,995-participant cluster-randomized trial in rural China showed that use of a potassium-enriched salt substitute significantly reduced stroke, major cardiovascular events, and mortality without increasing serious hyperkalemia, providing a robust evidence base for population-level sodium-reduction policies across Asia.^114^

Some Asian countries have demonstrated scalable, system-level solutions to improve medication affordability. For example, Sri Lanka’s centralized national procurement model,^115^ where a single government entity tenders and negotiates for all essential medicines, has markedly reduced costs and stabilized supply, illustrating how collective purchasing can strengthen access to cardiometabolic therapies.

**Figure 3:**
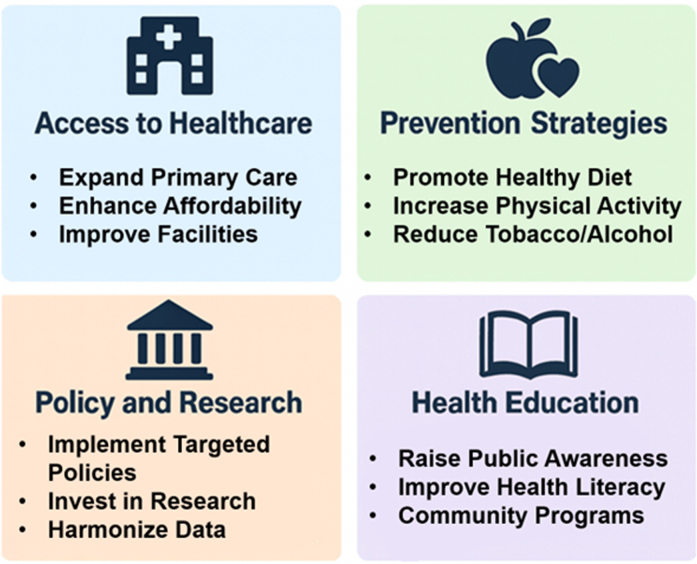
Strategic Framework to Address Global Inequities in Cardiometabolic Disease in Asia This framework outlines key system-level strategies to reduce inequities in cardiometabolic health worldwide. Four interconnected pillars are shown: 1) access to healthcare: expanding primary care, improving affordability, and strengthening facilities; 2) prevention strategies: promoting healthy diet, physical activity, and reduction of tobacco and alcohol use; 3) policy and research: implementing targeted national policies, investing in research, and harmonizing global data; and 4) health education: enhancing public awareness, health literacy, and community engagement. Together, these components highlight that sustainable progress in cardiometabolic health requires integrated action across healthcare delivery, prevention, policy, and education.

Improving cardiometabolic health will require cooperation across sectors, not only within the health system. Walkable cities, agricultural subsidies for fruit and vegetables, and workplace policies that promote movement all help lower metabolic risk.^116^ Education, particularly for girls and women, produces sustained downstream benefits through higher income, greater autonomy, and improved health literacy.^117^ Conditional cash-transfer programs, such as those implemented in Latin America, lowered obesity and hypertension within a decade, demonstrating that poverty reduction itself improves cardiometabolic health.^118^ Yet despite noncommunicable diseases accounting for more than 70% of global mortality, they receive <2% of international health aid.^119^ Redirecting even a small share of funding toward chronic-disease prevention could avert millions of premature deaths, including many across Asia (Figure 3).

### Therapeutic innovations

Breakthrough metabolic therapies have transformative relevance for Asia, with semaglutide 2.4 mg reducing major adverse cardiovascular events by 20% in the SELECT trial^120^ and tirzepatide achieving almost 20% weight loss alongside substantial cardiometabolic improvements.^121^ Dual-incretin agents have also shown roughly 70% reductions in new-onset diabetes in high-risk populations, signaling a paradigm shift in both prevention and treatment.^122^

Yet access to these therapies remains profoundly unequal. In high-income settings, annual treatment costs for glucagon-like peptide-1 receptor agonists exceed US$10,000 per patient, and insurance coverage is inconsistent. Across most low- and middle-income Asian countries, these agents are either unavailable or priced entirely out of reach.^123^ Achieving equitable access will require substantial price reductions, expanded insurance coverage, improved health literacy, and strategies to address limits on prescribing.^124^ Without fair licensing or affordable pricing models, therapies capable of materially reducing cardiometabolic deaths will remain inaccessible to the populations carrying the greatest burden.

Similar disparities exist for established cardiometabolic medications. Sodium–glucose cotransporter-2 inhibitors—shown to reduce heart failure and renal outcomes by 25%-35%—remain prohibitively expensive in many Asian regions.^125^ Statin coverage among individuals with clear indications reaches 66.5% globally, but in LMICs across Asia it remains as low as 3%-17%,^126^ illustrating the magnitude of therapeutic underuse.

Additionally, fixed-dose cardiovascular polypills can meaningfully improve adherence and reduce cardiovascular morbidity, as shown in both heart failure^127^ and post–myocardial infarction populations,^128^ but real-world uptake, particularly in resource-limited Asian settings, depends on addressing the current health-system constraints.

Finally, the integration of multimodality imaging markers, such as global longitudinal strain, coronary artery calcium, and myocardial tissue characterization, can potentially identify high-risk individuals years before clinical events occur.^129^ When combined with polygenic and metabolic risk scores, these tools offer a scalable pathway toward early, tailored intervention across diverse Asian populations.^130^

## Toward Prevention and Future Directions

Evidence from countries that combined economic policy, universal coverage, and community prevention shows that convergence is possible.^131^ When antihypertensive and statin coverage surpass 70% and tobacco use falls below 15%, cardiovascular mortality can decrease by more than half within 2 decades.^132^ Modelling suggests that extending these approaches globally could prevent up to 50 million premature deaths by 2050.^133^ At the individual level, intensive pharmacologic therapy, including statins, beta-blockers, angiotensin-converting enzyme inhibitors, and aspirin, for people with a 10-year Framingham risk ≥30% yields an estimated 11% reduction in major cardiovascular events.^134^

Ultimately, achieving cardiometabolic equity depends on society’s ability to translate medical advances into real, population-level benefits. The same forces that drive metabolic disease also undermine broader population health.^135^ Across Asia, uneven access to preventive care, essential therapies, and emerging treatments continues to determine who gains from progress and who is left behind.

Finally, artificial intelligence is rapidly advancing from simple diagnostic interpretation toward transformer-based multimodal systems.^136^ The combination of metabolomics and machine learning has shown promise in Asian populations in identifying ethnic-specific metabolic pathways,^137^ improving risk prediction, and developing tailored preventive strategies targeting cardiometabolic disease.

## Conclusions

Cardiometabolic disease reveals a growing gap between scientific progress and equitable benefit. As the burden shifts toward regions with limited health-system capacity, particularly across Asia, the challenge is implementation. Strengthening primary care, improving access to essential therapies, and integrating chronic disease management into universal health coverage are critical to closing this gap.

## Funding Support and Author Disclosures

Dr Butler has been a consultant for Abbott, Adaptyx, American Regent, Amgen, AskBio, AstraZeneca, Bayer, Boehringer Ingelheim, Boston Scientific, Bristol Myers Squibb, Cardiac Dimension, Cardior, CSL Vifor, CVRx, Cytokinetics, Daxor, Diastol, Edwards, Element Sciences, Faraday, Idorsia, Impulse Dynamics, Imbria, Innolife, Intellia, Inventiva, Levator, Lexicon, Eli Lilly, Mankind, Medtronic, Merck, New Amsterdam, Novartis, NovoNordisk, Pfizer, Pharmacosmos, Pharmain, Prolaio, Pulnovo, Regeneron, Renibus, Reprieve, Roche, Rycarma, Saillent, Salamandra, Salubris, SC Pharma, SQ Innovation, Secretome, Sequanna, Transmural, TekkunLev, Tenex, Tricog, Ultromic, Vera, and Zoll. Dr Tromp is supported by the National University of Singapore Start-up grant, and the CS-IRG from the National Medical Research Council; has received research support from AstraZeneca and consulting or speaker fees from Roche Diagnostics, the Asian Development Bank; and owns a patent US-10702247-B2 unrelated to the present work. All other authors have reported that they have no relationships relevant to the contents of this paper to disclose.
